# Supplementary material for: Modular subgraphs in large-scale connectomes underpin spontaneous co-fluctuation events in mouse and human brains
Source: Commun Biol. 2024 Jan 24;7:126. doi: 10.1038/s42003-024-05766-w (PMC10810083; doi:10.1038/s42003-024-05766-w)
Supplement: Supplementary file 2 — Supplementary Figures [file 42003_2024_5766_MOESM2_ESM.pdf]

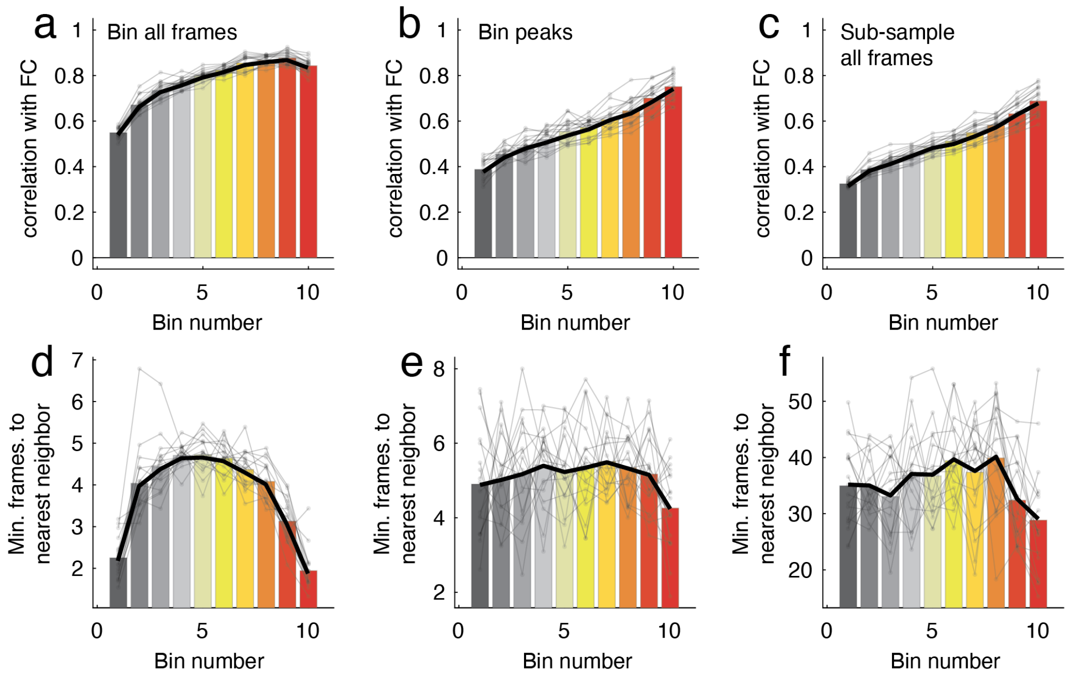

Figure S1. **Effect of RMS amplitude and sampling strategy on predicted FC.** In the main text we predicted FC using select subsets of frames. Here, subdivide frames into percentile bins based on their RMS. We explore several binning strategies. (a) First we partition all frames from each scan into deciles. As in Cutts *et al.* [16], Ladwig *et al.* [26], we observe a near-monontonic increase in correlation but also find that the second highest amplitdue bin exhibits a marginally greater correspondence with FC. (b) The second strategy for binning involves first selecting peak frames of the RMS signal but otherwise divides the frames into RMS deciles. Here, we find a true monotonic increase in RMS across bins. (c) We can also use the bins defined in a and subsample a number of frames equal to a much smaller number–in this case, the number of peaks per bin from b. What explains why a exhibits a peak in the second to last bin while strategies b and c exhibit monotonic increases? Strategy a bins all frames. Given the strong autocorrelation in fMRI BOLD data and the fact that events take longer to unfold relative to lower-amplitude peaks [18], the highest bin tends to include many temporally proximal frames. We quantify the relative "nearness" of frames to one another by calculating for each frame assigned to a given bin its nearest neighbor and then averaging those values. When we plot these values for each bin, we find that strategy a yields an inverted U-shaped curve, suggesting that the highest (and lowest) amplitude bins are composed of more temporally contiguous frames than middle-amplitude frames and therefore may not effectively sample the entire time series, introducing a bias (see d). In contrast, strategies b and c, which sample peaks and a small number of random samples, exhibit near-uniform nearest neighbor curves, suggesting that if a bias exists, it may be less severe (panels e and f).

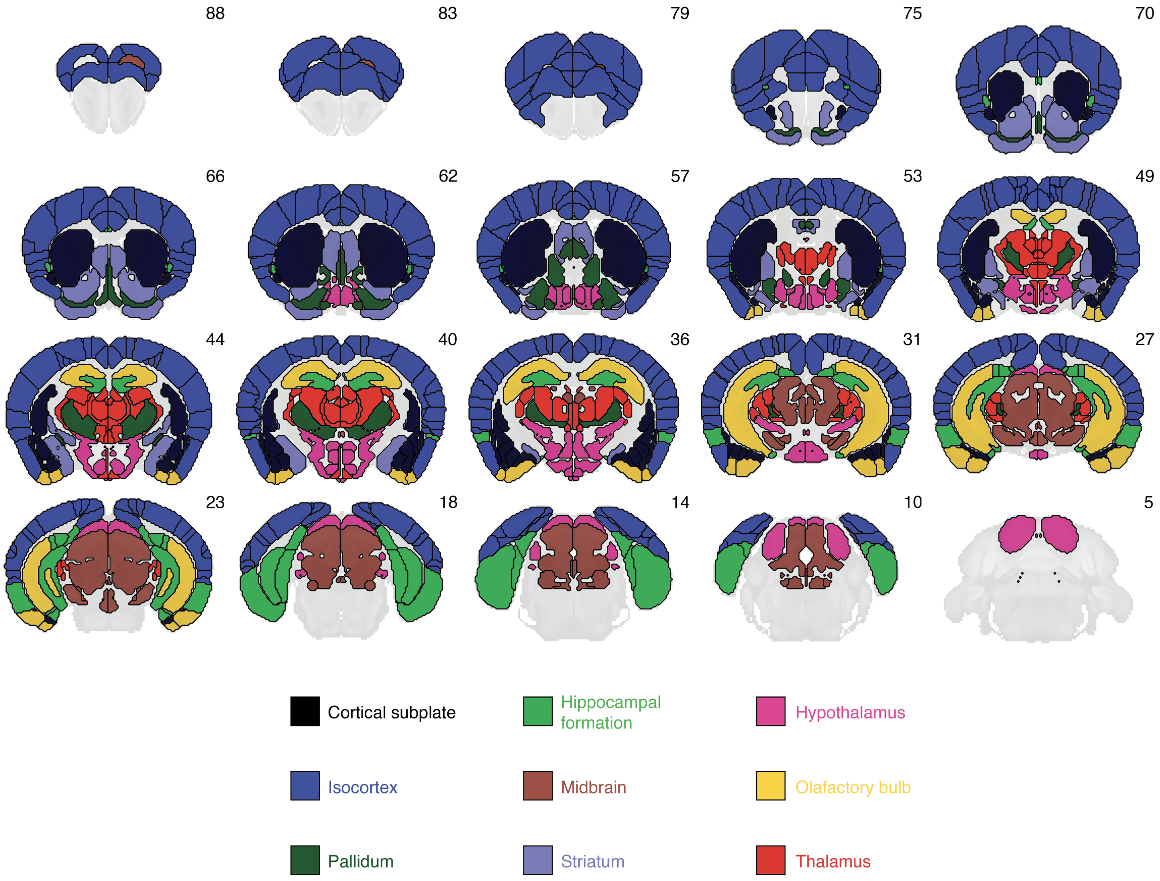

Figure S2. **Macroscopic system labels based on anatomy.** .

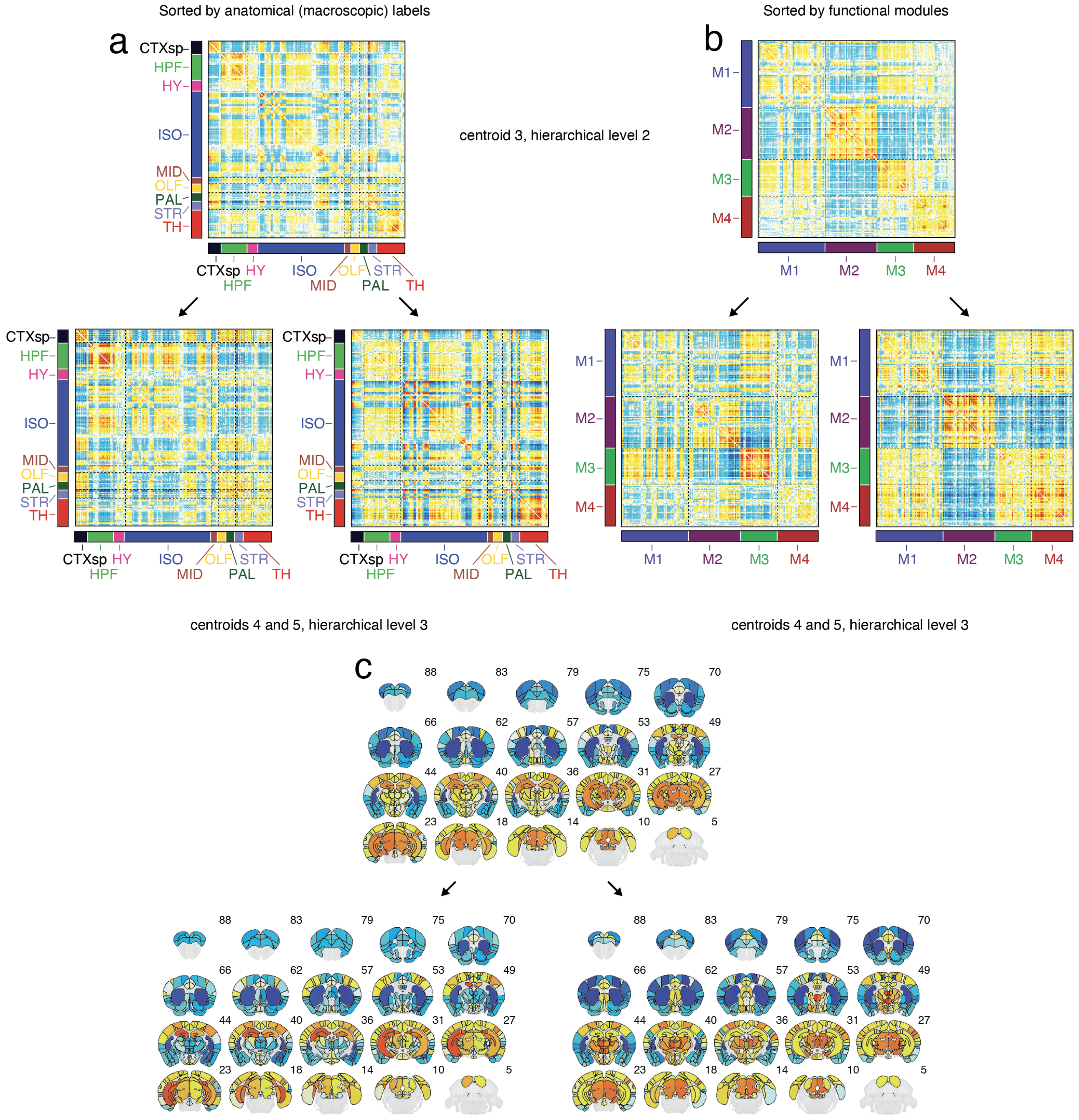

Figure S3. **Sub-divisions of event cluster 3.** Event cluster 3 in hierarchical level 2 gets subdivided into two clusters at hierarchical level 3 (labeled clusters 4 and 5). (*a*) Cluster centroids ordered by anatomical system labels. (*b*) Cluster centroids ordered by functional systems. (*c*) Leading eigenvectors for each cluster centroid projected onto brain volume.

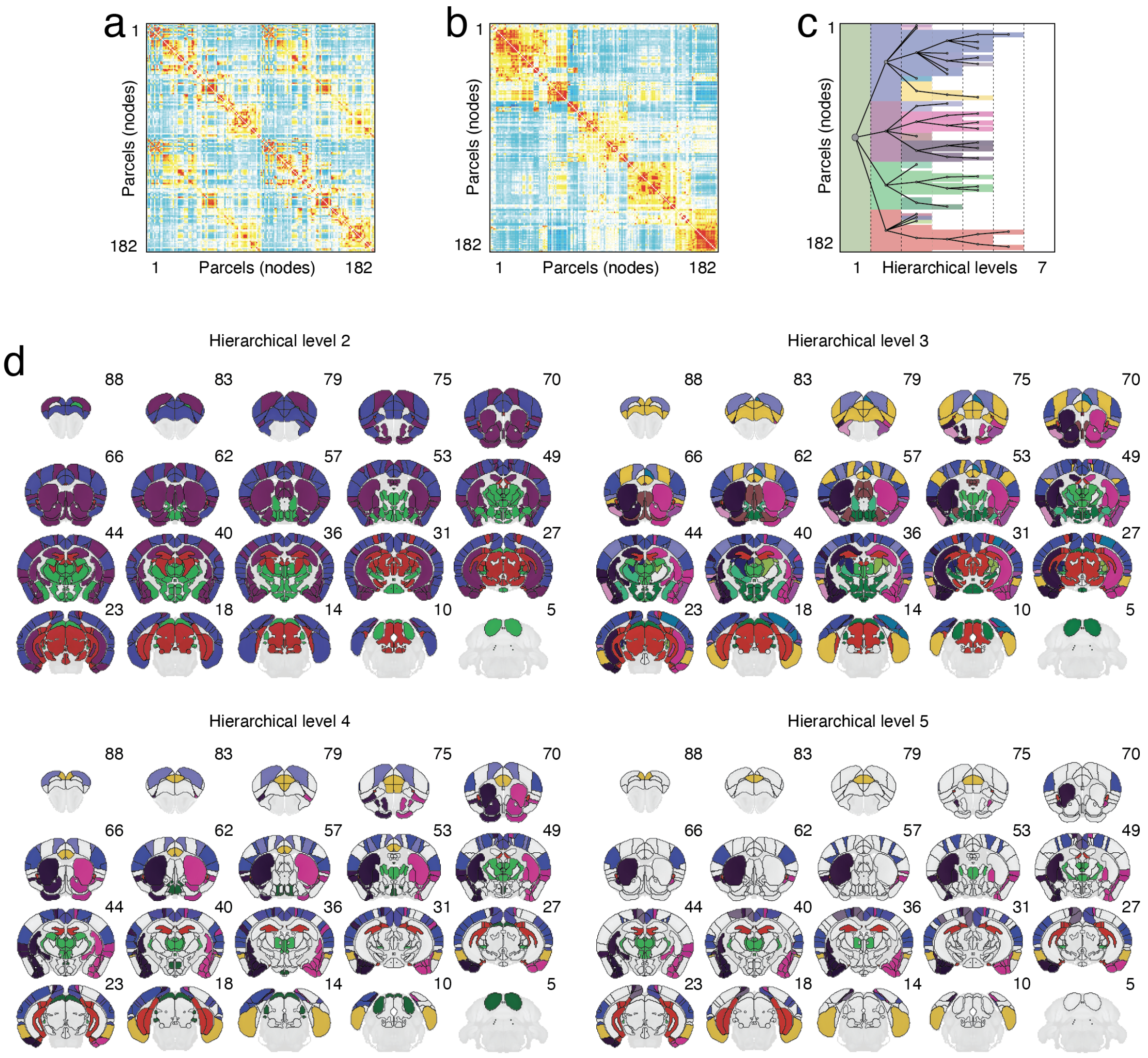

Figure S4. **Hierarchical decomposition of static FC into brain systems.** (*a*) Unsorted FC matrix. (*b*) Optimally sorted FC matrix. (*c*) Hierarchical community labels and dendrogram. (*d*) Functional system labels at different hierarchical levels.

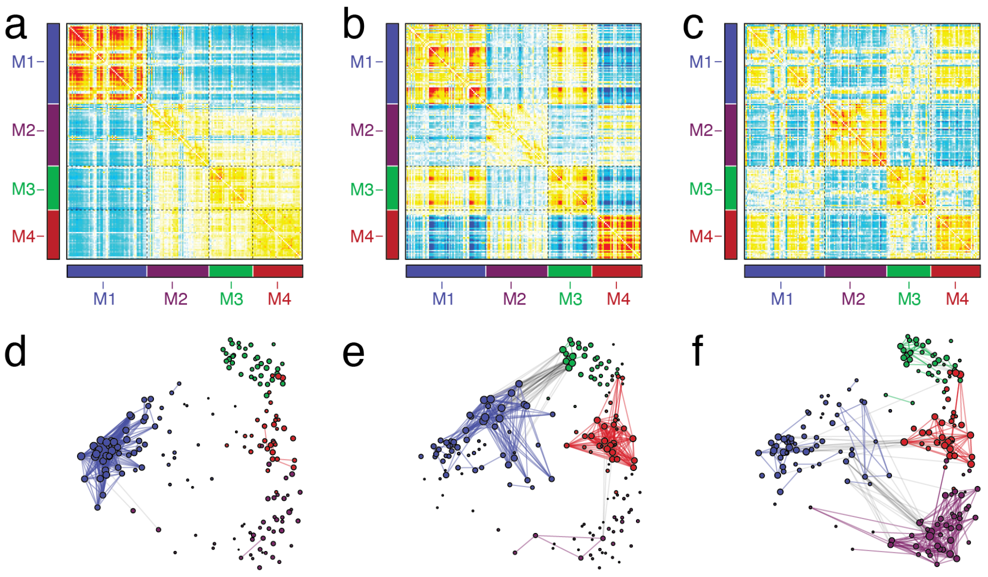

Figure S5. **Event co-fluctuation patterns at coarse scale.** Panels *a*-*c* depict the same co-fluctuation patterns shown in Fig. 3. However, here we sort rows and columns by functional system labels. Panels *d*-*f* depict co-fluctuation matrices thresholded at 2.5% sparsity. Nodes are colored based on the functional system label. Layout (nodal coordinates) was determined by force-directed algorithm applied to fully-weighted connectome. Note that the aim of this figure is to reinforce the idea that the strongest co-fluctuations (edges) in each cluster centroid tend to fall within functional modules.

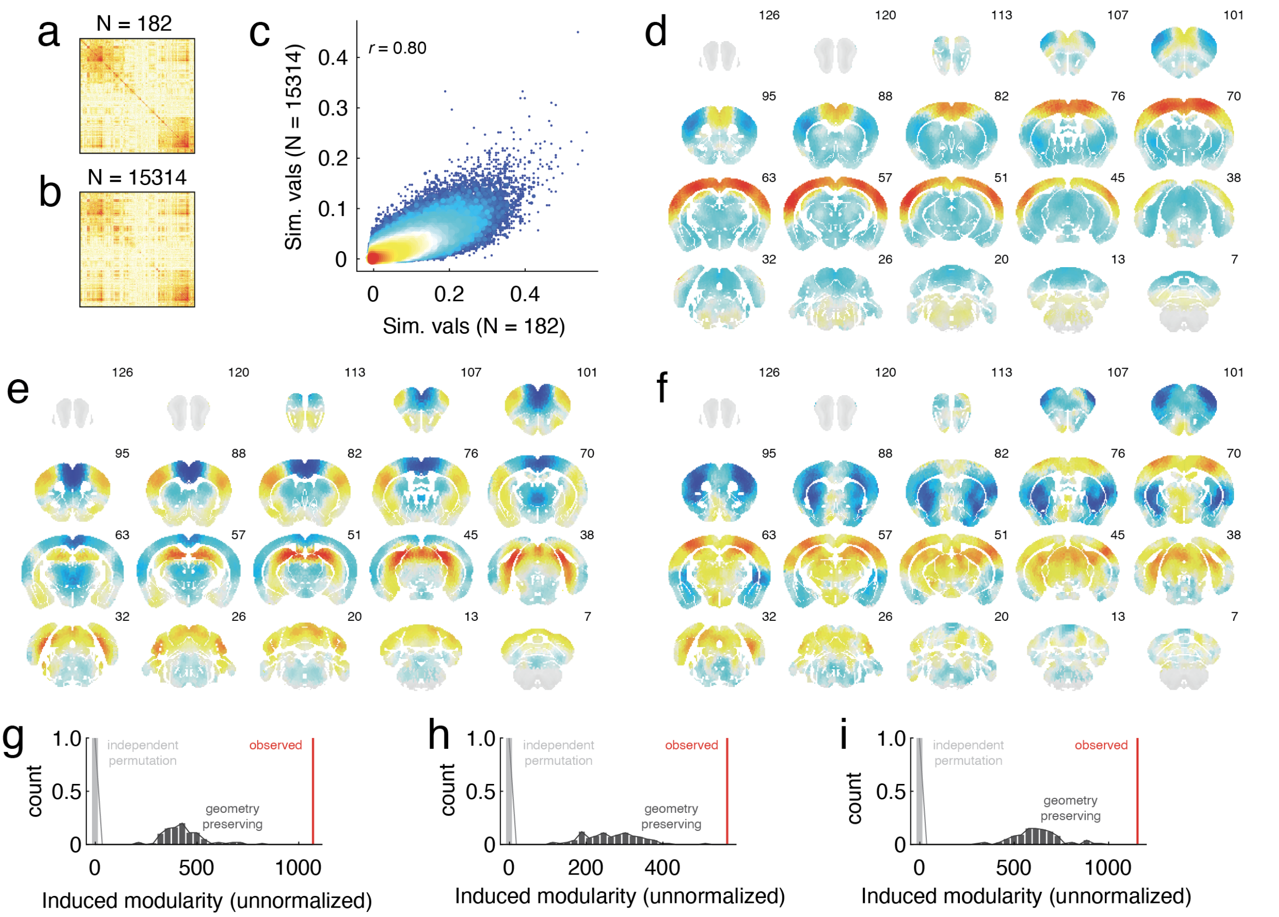

Figure S6. **Voxel-level replication of main results.** Similarity between pairs of events for parcellated data from main text (*a*) and voxel-wise data (*b*) derived from Coletta *et al.* [100]. (*c*) Scatterplot of upper triangle elements from panels *a* and *b*. Panels *d*, *e*, and *f* show cluster centroids 1-3 from the main text but at voxel resolution. Panels *d*-*f* show induced modularity of bipartitions compared to permutation-based null model. Note that here, structural connectivity is defined based on the fully-weighted (unthresholded) connectome described by Coletta *et al.* [100].

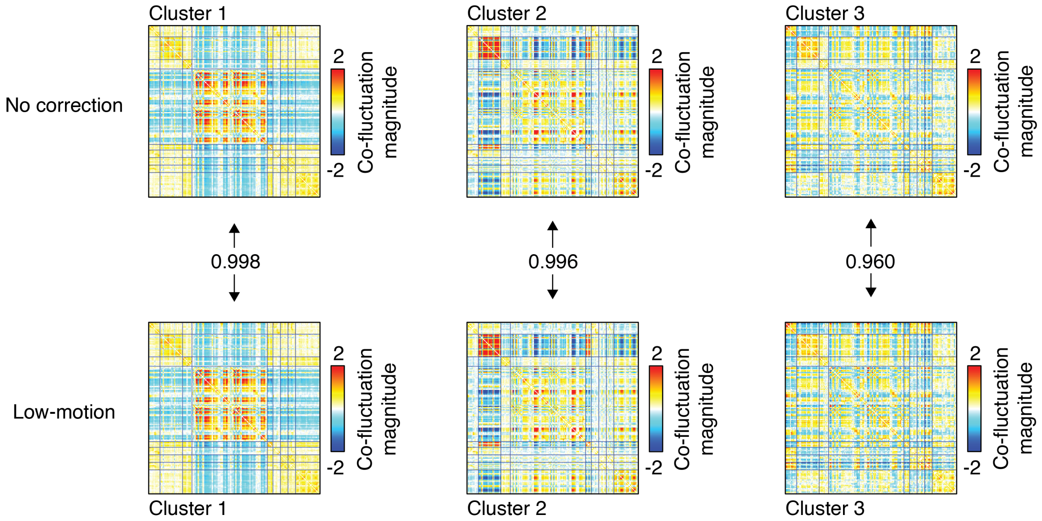

Figure S7. **Assessing impact of in-scanner motion on event clusters.** In the main text we detected high-amplitude events, which we subsequently clustered and linked to the modular structure of anatomical connectivity. Although the fMRI data were processed using a pipeline that include denoising and motion correction steps, it is possible that residual motion impacted event detection so that motion spikes were coincident with event timing (though note that the mouse data exhibit a relatively low level of in-scanner motion from the outset; mean±s.d. framewise displacement of 0.031 ± 0.015). To address this concern, we repeated event detection and clustering after including a more conservative motion screening procedure. Briefly, we excluded from analysis any frame whose framewise displacement exceeded a threshold of 0.075 mm. We also excluded frames within five samples of a high-motion frames and dropped any low-motion frames that were part of short contiguous sequences (fewer than ten low-motion frames in series). With the exception of one animal for whom 417 frames (approximately 27%) were flagged as high-motion, the mean number of frames discarded was 47.5 (range of 0 to 78 frames). This event detection procedure identified 641 events as opposed the 624 originally reported. The hierarchical clustering procedure identified three large clusters at the second level, comprising 39.5%, 31.2%, and 8.0% of all events. These clusters, whose centroids shown above in the bottom row next to the label "Low-motion" exhibited a near perfect correspondence with the clusters reported in the main text (labeled "No correction"). These cluster centroids get propagated to the next analysis, in which they are linked to anatomical connectivity. Because of their near-perfect correspondence with the originally reported cluster centroids, they will induce almost identical levels of modularity, replicating the results in the main text.

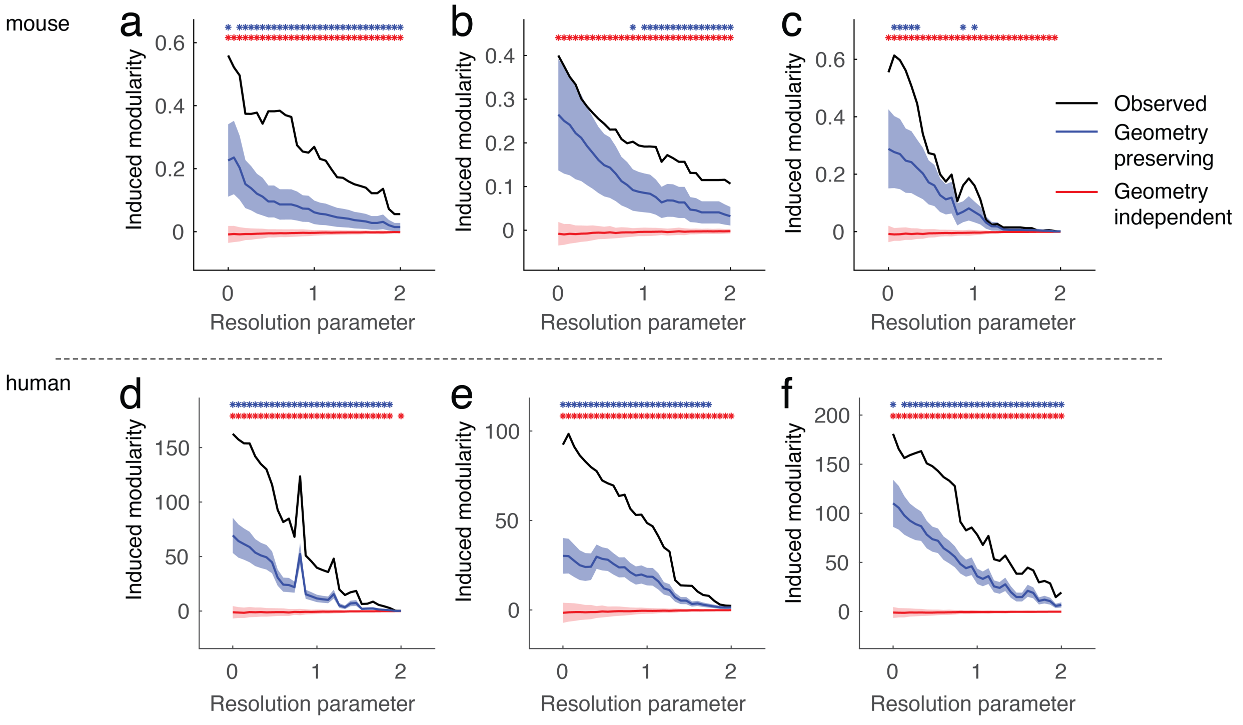

Figure S8. **Parametric variation in induced modularity.** In the main text we calculated the modularity of a subgraph induced by a bipartition derived from event cluster centroids. Here, we explore the effect of varying the threshold parameter used to define the two clusters. Panels *a*-*c* show the induced modularity of the observed subgraph compared against the modularity estimated under two null models – one that preserves geometry (Geom.) and another that does (Ind.). Blue and red stars indicate statistical significance (critical p-value adjusted to maintain a false discovery rate of 5%). Panels *d*-*f* show analogous plots for the human imaging data.

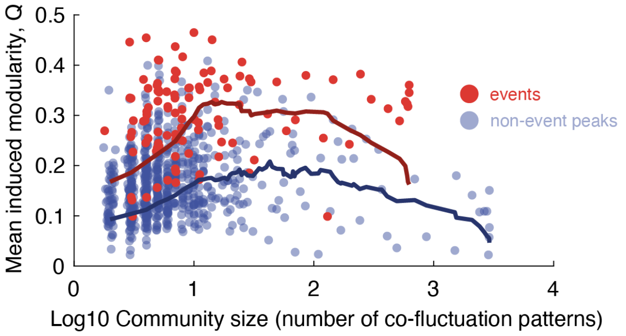

Figure S9. **Induced modularity for events *versus* non-event peaks.** In the main text we showed that the high-amplitude events are undergirded by a highly modular bipartition of the anatomical network. Here, we show that, the induced modularity of that network is greater than networks of equal size but corresponding to non-event peaks. Specifically, we calculated the mean induced modularity for all subnetworks of size N_s. We repeated this procedure for both the events (red) and non-event peaks (blue). We used functional data analysis to compare the two curves. Specifically, we calculated the summed difference between each point. We compared this value against a null model in which we randomly assigned event and non-event centroids to either class. We found that the observed difference in curves was statistically greater than that of the null distribution ($p < 10^{-2}$).

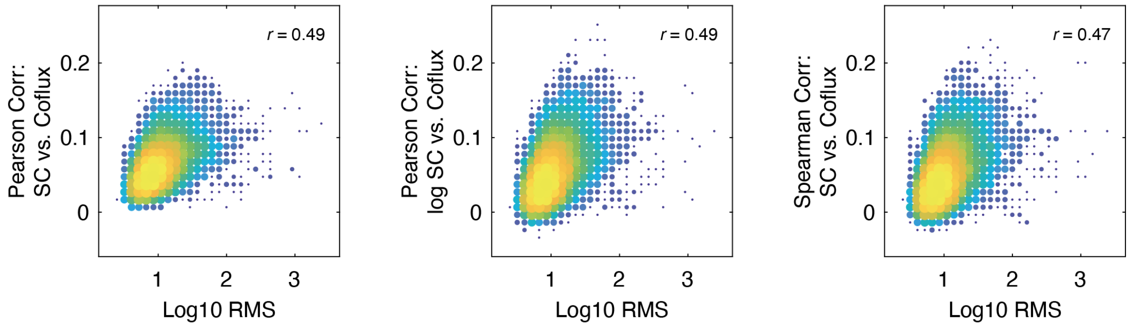

Figure S10. **Framewise structure-function correlations scale with RMS.** At each frame, we calculated the correlation between SC and the co-fluctuation matrix. We calculated three separate versions of the correlation coefficient. First, we calculated the bivariate product-moment correlation between the raw SC weights and co-fluctuation amplitudes. We also calculated the bivariate correlation using log-transformed SC weights. Finally, we calculated the rank correlation between SC weights and co-fluctuation amplitudes. We compared these frame-resolved structure-function correlation coefficients with the RMS amplitude of global co-fluctuations at each frame. In all cases, we found that structure-function correlation was strongest during high-amplitude frames.

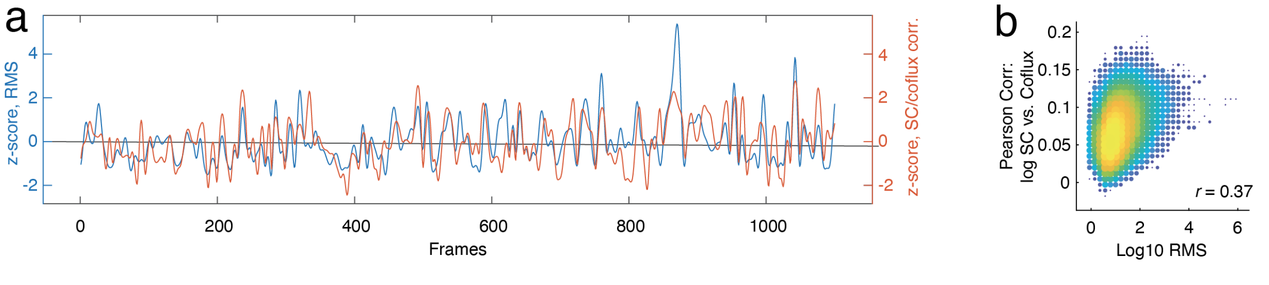

Figure S11. **Framewise structure-function correlations scale with RMS using human data from HCP.** (*a*) Plot showing example z-scored RMS and correlation coefficient between structural edge weights and corresponding elements of the time-varying co-fluctuation matrix. (*b*) Two-dimensional histogram of the RMS *versus* correlation.
