# Supplementary material for: Modular subgraphs in large-scale connectomes underpin spontaneous co-fluctuation events in mouse and human brains
Source: Commun Biol. 2024 Jan 24;7:126. doi: 10.1038/s42003-024-05766-w (PMC10810083; doi:10.1038/s42003-024-05766-w)
Supplement: Supplementary file 3 — Description of Additional Supplementary Files [file 42003_2024_5766_MOESM3_ESM.pdf]

Description of Additional Supplementary Files

File name: Supplementary Data 1
Description: Data used to make the plots in Figure 2.

File name: Supplementary Data 2
Description: Data used to make the plots in Figure 3.

File name: Supplementary Data 3
Description: Data used to make the plots in Figure 4.

File name: Supplementary Data 4
Description: Data used to make the plots in Figure 5.
