# Supplementary material for: Modular subgraphs in large-scale connectomes underpin spontaneous co-fluctuation events in mouse and human brains
Source: Commun Biol. 2024 Jan 24;7:126. doi: 10.1038/s42003-024-05766-w (PMC10810083; doi:10.1038/s42003-024-05766-w)
Supplement: Supplementary file 8 — Reporting Summary [file 42003_2024_5766_MOESM8_ESM.pdf]

nature portfolio

Corresponding author(s): Richard Betzel

Last updated by author(s): Dec 11, 2023

Reporting Summary

Nature Portfolio wishes to improve the reproducibility of the work that we publish. This form provides structure for consistency and transparency in reporting. For further information on Nature Portfolio policies, see our Editorial Policies and the Editorial Policy Checklist.

Statistics

For all statistical analyses, confirm that the following items are present in the figure legend, table legend, main text, or Methods section.

n/a	Confirmed	
☐	☒	The exact sample size (n) for each experimental group/condition, given as a discrete number and unit of measurement
☐	☒	A statement on whether measurements were taken from distinct samples or whether the same sample was measured repeatedly
☐	☒	The statistical test(s) used AND whether they are one- or two-sided<br>*Only common tests should be described solely by name; describe more complex techniques in the Methods section.*
☐	☒	A description of all covariates tested
☐	☒	A description of any assumptions or corrections, such as tests of normality and adjustment for multiple comparisons
☐	☒	A full description of the statistical parameters including central tendency (e.g. means) or other basic estimates (e.g. regression coefficient) AND variation (e.g. standard deviation) or associated estimates of uncertainty (e.g. confidence intervals)
☐	☒	For null hypothesis testing, the test statistic (e.g. F, t, r) with confidence intervals, effect sizes, degrees of freedom and P value noted<br>*Give P values as exact values whenever suitable.*
☒	☐	For Bayesian analysis, information on the choice of priors and Markov chain Monte Carlo settings
☒	☐	For hierarchical and complex designs, identification of the appropriate level for tests and full reporting of outcomes
☐	☒	Estimates of effect sizes (e.g. Cohen's d, Pearson's r), indicating how they were calculated

Our web collection on statistics for biologists contains articles on many of the points above.

Software and code

Policy information about availability of computer code

Data collection	Human data used in this study is publicly available. The authors of this study did not write custom code to collect any of this data; therefore the authors did not use software for data collection.
Data analysis	Human Connectome Project data are provided already minimally preprocessed at the ConnectomeDB (https:/db.humanconnectome.org/app/template/Login.vm). Subject specific parcellations were fit with FreeSurfer 6.0.1 using code available here: https://github.com/faskowit/multiAtlasTT and data available here: https://figshare.com/articles/multiAtlasTT_data_hcptrained/7552853. fMRI data were nuisance regressed with code available here: https://github.com/faskowit/app-fmri-2-mat which uses Nilearn's signal.clean, from Nilearn 0.5.0.

For manuscripts utilizing custom algorithms or software that are central to the research but not yet described in published literature, software must be made available to editors and reviewers. We strongly encourage code deposition in a community repository (e.g. GitHub). See the Nature Portfolio guidelines for submitting code & software for further information.

Data

Policy information about availability of data

All manuscripts must include a data availability statement. This statement should provide the following information, where applicable:

- Accession codes, unique identifiers, or web links for publicly available datasets
- A description of any restrictions on data availability
- For clinical datasets or third party data, please ensure that the statement adheres to our policy

This study analyzes mouse and human imaging datasets. The human imaging data come from the Human Connectome Project. Access to raw and minimally processed data can be obtained by digitally signing a data use agreement (https://db.humanconnectome.org/app/template/Login.vm). Mouse structural connectivity data were derived from a voxel-scale model of the mouse connectome and made available by the Allen Brain Institute (https://data.mendeley.com/datasets/dxtzpvv83k/2). Mouse functional imaging data are publicly available (https://data.mendeley.com/datasets/7y6xr753g4/1).

Research involving human participants, their data, or biological material

Policy information about studies with human participants or human data. See also policy information about sex, gender (identity/presentation), and sexual orientation and race, ethnicity and racism.

Reporting on sex and gender	Neither sex nor gender were considered in our analyses; imaging and network data were pooled across the entire cohort.
Reporting on race, ethnicity, or other socially relevant groupings	No racial, ethnic, or social groupings were analyzed; imaging and network data were pooled across the entire cohort.
Population characteristics	Of the participants studied here, 56% were female.
Recruitment	HCP subjects were recruited from the Missouri Department of Health and Senior Services Bureau of Vital Records. NKI subjects were recruited based on zip code (e.g. advertisement flyer mailings, posting of recruitment materials in local shops and meeting places). The authors of the present study did not collect any of the primary imaging data nor did they conduct recruitment of subjects. Therefore, the authors are not knowledgeable about possible self-section biases or other biases associated with the data collection of these two cohorts. We would estimate that potential biases related to recruitment would not likely impact the main results of the study.
Ethics oversight	HCP was approved by the Washington University Institutional Review Board. NKI was approved by the Institutional review boards at Nathan Kline Institute and at Montclair State University.

Note that full information on the approval of the study protocol must also be provided in the manuscript.

Field-specific reporting

Please select the one below that is the best fit for your research. If you are not sure, read the appropriate sections before making your selection.

☒ Life sciences ☐ Behavioural & social sciences ☐ Ecological, evolutionary & environmental sciences

For a reference copy of the document with all sections, see nature.com/documents/nr-reporting-summary-flat.pdf

Life sciences study design

All studies must disclose on these points even when the disclosure is negative.

Sample size	The mouse functional imaging data came from a previously collected dataset comprising N = 20 mice.<br><br>The Human Connectome Project (HCP) aimed to collect healthy adult twins, ages 22-25 years old (Van Essen, 2012). The definition of "healthy" was broad, in order to collect a sample representative of the United States population in terms of behavior, ethnic, and socioeconomic diversity. In this study, a provided subset of subjects called the "Unrelated 100" was used.
Data exclusions	Participants were excluded based on motion and data quality criteria.<br><br>For our human neuroimaging data, data exclusions were based on data quality, to filter out scans with excessive motion or image artifact, and based on data completeness, to ensure that each subject that the appropriate meta-data, and both structural and functional scans. For HCP, subjects were considered for data exclusion based on the mean and mean absolute deviation of the relative root-mean-square motion across either four resting-state MRI scans (file: Movement_RelativeRMS.txt) or one diffusion MRI scan (file: eddy_unwarped_images.eddy_movement_rms), resulting in four summary motion measures. If a subject exceeded 1.5 times the interquartile range (in the adverse direction) of the measurement distribution in two or more of these measures, the subject was excluded. These exclusion criteria were established before the current study. Four subjects were excluded based on these criteria. One subject was excluded for software error during diffusion MRI processing. For NKI, subjects were considered for data exclusion based on having a complete set of T1w, resting state, diffusion images, and meta-data, as well as the quality of the aforementioned images. The ENIGMA QC FreeSurfer tools

(http://enigma.ini.usc.edu/protocols/imaging-protocols/), MRIQC (https://mriqc.readthedocs.io/en/stable/), eddy_qc (https://fsl.fmrib.ox.ac.uk/fsl/fslwiki/eddyqc/UsersGuide), and QAscripts (https://www.med.upenn.edu/cmroi/qascripts.html) were used to derive image quality metrics to assess data quality of the T1w, resting state, and diffusion images. Details of exclusion criteria can be found in the manuscript section "Quality Control".

For mouse imaging data, we performed frame censoring to remove high motion frames. We further excluded frames that were temporally proximal to the high motion frames. Finally, we excluded frames that failed to form long contiguous sequences of low motion.

Replication	The primary effects--detection of low-dimensional set of high-amplitude network states and correspondence of structural modules with co-fluctuation patterns--replicated across mouse and human data and were reported in the manuscript.
Randomization	No comparisons made. Data were treated as representative samples.
Blinding	No comparisons made. Data were treated as representative samples.

Reporting for specific materials, systems and methods

We require information from authors about some types of materials, experimental systems and methods used in many studies. Here, indicate whether each material, system or method listed is relevant to your study. If you are not sure if a list item applies to your research, read the appropriate section before selecting a response.

Materials & experimental systems

n/a	Involved in the study	
☒	☐	Antibodies
☒	☐	Eukaryotic cell lines
☒	☐	Palaeontology and archaeology
☐	☒	Animals and other organisms
☒	☐	Clinical data
☒	☐	Dual use research of concern
☒	☐	Plants

Methods

n/a	Involved in the study	
☒	☐	ChIP-seq
☒	☐	Flow cytometry
☐	☒	MRI-based neuroimaging

Animals and other research organisms

Policy information about studies involving animals; ARRIVE guidelines recommended for reporting animal research, and Sex and Gender in Research

Laboratory animals	Mouse: Chd8+/– transgenic mouse line
Wild animals	n/a
Reporting on sex	6 males, 13 females
Field-collected samples	n/a
Ethics oversight	All in vivo experiments were conducted in accordance with the Italian law (DL 26/214, EU 63/2010, Ministero della Sanità, Roma) and the recommendations in the Guide for the Care and Use of Laboratory Animals of the NIH

Note that full information on the approval of the study protocol must also be provided in the manuscript.

Magnetic resonance imaging

Experimental design

Design type	Resting state (mouse). In this study, we used T1-weighted, resting state (fixation cross, eyes open), and diffusion weighted MRI scans (human).
Design specifications	No blocks were used in this study for these image acquisitions.
Behavioral performance measures	n/a

Acquisition

Imaging type(s)	structural, functional, diffusion
Field strength	3T
Sequence & imaging parameters	For HCP, a comprehensive description of the imaging parameters and image prepocessing can be found in Glasser et al. 2013. Images were collected on a 3T Siemens Connectome Skyra with a 32-channel head coil. Subjects underwent two T1-weighted structural scans, which were averaged for each subject (TR = 2400 ms, TE = 2.14 ms, flip angle = 8◦ , 0.7 mm isotropic voxel resolution). Subjects underwent four resting state fMRI scans over a two-day span. The fMRI data was acquired with a gradient-echo planar imaging sequence (TR = 720 ms, TE = 33.1 ms, flip angle = 52◦ , 2 mm isotropic voxel resolution, multiband factor = 8). Each resting state run duration was 14:33 min, with eyes open and instructions to fixate on a cross. Finally, subjects underwent two diffusion MRI scans, which were acquired with a spin-echo planar imaging sequence (TR = 5520 ms, TE = 89.5 ms, flip angle = 78◦ , 1.25 mm isotropic voxel resolution, b-vales = 1000, 2000, 3000 s/mm2 , 90 diffusion weighed volumes for each shell, 18 b = 0 volumes). These two scans were taken with opposite phase encoding directions and averaged.<br><br>For mouse, animal preparation, image data acquisition, and image data preprocessing for rsfMRI data have been described in greater detail elsewhere (Gutierrez et al 2019, Current Biology). Briefly, rsfMRI data were acquired on a 7.0-T scanner (Bruker BioSpin, Ettlingen) equipped with BGA-9 gradient set, using a 72-mm birdcage transmit coil, and a four-channel solenoid coil for signal reception. Single-shot BOLD echo planar imaging time series were acquired using an echo planar imaging sequence with the following parameters: repetition time/echo time, 1200/15 ms; flip angle, 30 degree; matrix, 100 ×100; field of view, 2 × 2 cm2; 18 coronal slices; slice thickness, 0.50 mm; 1500 (n = 19) volumes; and a total rsfMRI acquisition time of 30 min.
Area of acquisition	Whole-brain

Diffusion MRI ☒ Used ☐ Not used

Parameters	HCP: 3 shells (bvals: 1000, 2000, 3000), 90 directions per shell, 18 unweighted volumes; NKI: 1 shell (bval: 1500), 128 directions, 9 unweighted volumes

Preprocessing

Preprocessing software	Functional images in the HCP were processed with the HCP pipelines and downloaded after the 1200 subject data release. The HCP pipelines utilize FSL, Connectome Workbench, and custom MATLAB (pipelines available here: https://github.com/Washington-University/HCPpipelines; and describe in Glasser et al. 2013). Functional images in the NKI dataset were preprocessed using fMRIPrep 1.1.8, which is based on Nipype. Internal operations of fMRIPrep use Nilearn 0.5.0, ANTs 2.1.0, FreeSurfer 6.0.1, FSL 5.0.9, and AFNI v16.2.07. For more details about the pipeline, see the section corresponding to workows in fMRIPrep's documentation, for version 1.1.8 (https://fmriprep.org/en/stable/citing.html). Diffusion images were preprocessed following the DESIGNER protocol (Ades-Aron et al., 2018) which uses MRTrix 3.0 functions. To perform white matter model fitting and streamline tractography we used Dipy 0.16.0.
Normalization	Within the fMRIPrep workflow, ANTs is used to align functional images to the MNI Asymmetrical template version 2009c. For HCP, FSL FNIRT is used to align functional images in the FSL MNI template and furthermore, multi-modal registration is used to align surface functional data to the fs_LR surface space.
Normalization template	For HCP, fMRI data was analyzed after linear alignment (AC-PC) to the FSL MNI template.
Noise and artifact removal	For functional data of the HCP and NKI, we employed a 36-parameter nuisance regression strategy described Satterthwaite et. al (2013) and shown to be a relatively effective strategy (with and without spike regression) in Parkes et. al (2018). For diffusion images of the HCP, data is provided preprocessed.
Volume censoring	We used spike regression to preform volume censoring. For NKI, spike regressor was added for each fMRI frame exceeding 0.5 mm framewise displacement. For HCP, spike regression was not applied.

Statistical modeling & inference

Model type and settings	n/a
Effect(s) tested	n/a

Specify type of analysis: ☒ Whole brain ☐ ROI-based ☐ Both

Statistic type for inference<br>(See Eklund et al. 2016)	n/a
Correction	n/a

Models & analysis

n/a	Involved in the study
☐	☒ Functional and/or effective connectivity
☐	☒ Graph analysis
☒	☐ Multivariate modeling or predictive analysis

Functional and/or effective connectivity	Correlation
Graph analysis	Modularity maximization; weighted and directed mouse connectome; weighted and undirected human connectome.
